# The Diploic Veins: A Comprehensive Review with Clinical Applications

**DOI:** 10.7759/cureus.4422

**Published:** 2019-04-09

**Authors:** Stefan Lachkar, Mary-Margaret Dols, Basem Ishak, Joe Iwanaga, R. Shane Tubbs

**Affiliations:** 1 Anatomy, Seattle Science Foundation, Seattle, USA; 2 Neurosurgery, Seattle Science Foundation, Seattle, USA; 3 Medical Education and Simulation, Seattle Science Foundation, Seattle, USA

**Keywords:** diploic veins, diploic space, arteriovenous fistula, cerebrospinal fluid, diploic venous system

## Abstract

The diploic veins serve as an important connection between the extracranial and intracranial venous systems. They change in size during growth from adolescence to adulthood. The diploic space has been identified as an additional site of reabsorption of cerebrospinal fluid (CSF). Herein, the anatomy and physiology of the diploic veins are reviewed.

## Introduction and background

**Figure 1:**
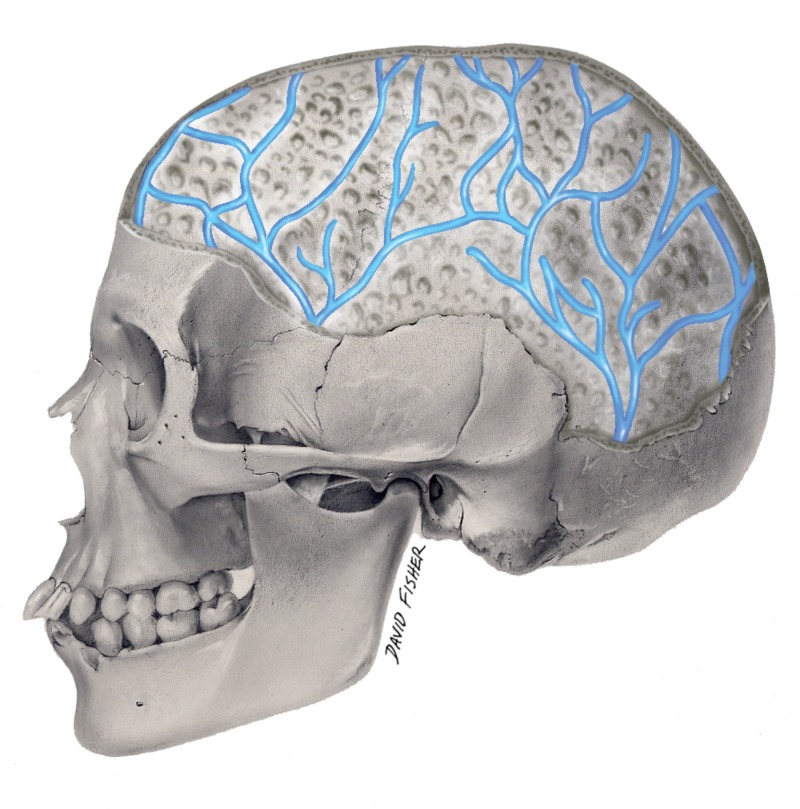
Schematic drawing of the human diploic veins from a lateral view

Diploic veins are valveless channels traveling between the inner and outer tables of the calvaria in the diploic space (diploë) (Figure 1). These veins were first identified by Guillaume.

Dupuytren in 1803 [1], and since then have been ill-defined and often misrepresented in the literature [2-4]. They consist of larger veins communicating with an extensive network lined by a single epithelial layer dispersed throughout the calvaria. This has made them difficult to access and therefore to study [5]. Also, diploic veins are inconsistently identified by traditional neurosurgical imaging techniques and can be involved in a wide range of pathological and surgical considerations [6].

Five patterns have been used to describe the diploë in the parietal bone: spider, serpentine, coronal, vine/bonsai, and thousand lakes types [1]. Vine/bonsai, found to be the most common, is defined as a single vertical channel on the posterior aspect of the parietal bone that extends upwards, narrowing and flowing towards the frontal bone as it branches out. It is contiguous with the transverse-sigmoid sinuses. Most of these patterns are characterized by directly draining to the sphenoparietal sinus and contain major branches visible to the naked eye. Diploic veins of the frontal bone are constituted of either one or two channels accompanied with minor braches in the midfrontal region. The minor channels of the frontal diploic veins (FDVs) may unite with the superior orbital vein. Diploic veins of the occipital bone contribute minutely to venous drainage and therefore do not illustrate any clear patterning [1].

In adults, diploic veins are most common, and articulate most often, in the middle rather than anterior or posterior portions of the skull [7]. However, there is almost always symmetry across the hemicrania regardless of distribution pattern [8]. Diploic veins in the frontal bone are more developed during adolescence, as peak growth of the frontal sinus is supported by increased vascularity [9]. During the transition from adolescence to adulthood, diploic veins are mostly observed in parietal bone [10]. Considering the differences in anatomical features between adolescents and adults, there are likely to be significant changes in the network of diploic veins over the lifespan. This can lead to important distinctions in the diagnosis and treatment of extra- and intra-cranial infections between these two groups. For example, diploic veins in the frontal bone are often related to Pott’s puffy tumor. Infection of the frontal sinus spreads through the diploë either to the subperiosteal space or to subdural/subarachnoid spaces via valveless flow [11].

In adults, the diploic veins can be divided into three regions: the FDV with its major drainage near the supraorbital notch/foramen, the anterior temporal diploic vein (ATDV) draining near the pterion, and the posterior temporal diploic vein (PTDV) with drainage near the asterion [5]. When present, the FDV runs near the superolateral aspect of the frontal sinus and communicates with the superior sagittal sinus. The PTDV also communicates with the superior sagittal sinus and connects the extracranial and intracranial veins through communication with emissary veins. Pterional, supraorbital, and modified orbito-zygomatic craniotomies can all communicate with the FDV and ATDV [3]. Bleeding during these procedures is often from the diploë where there is a risk for air embolism [5]. Although most bony bleeding from the diploic space is easily controlled with bone wax [12], it can be useful to plan neurosurgical procedures around the major drainage of the diploic systems [13-14].

Tsutsumi [6] proposed an alternative classification of the diploic veins into four pathways: pteriofrontoparietal (PFP), frontoorbital (FO), occipitoparietal (OP), and occipitocervical (OC). The PFP lies in the parietal region and aids in connecting the sphenoparietal sinus lateral edge to the superior sigmoid sinus. The PFP consists of a singular trunk arising from the pterional region and divides into further branches at the frontal eminence until the parietal straight sigmoid sinus is reached. Two major components of the FO are the orbital and pterional parts. As the FO portions and PFP merge with the sphenoparietal sinus proximally, it descends further to continue as the middle meningeal vein, coursing beside the foramen ovale and draining into the pterygoid plexus. As a single trunk coursing in the superiorinferior direction, the OP links the connection point of the transverse-sigmoid sinus to the superior sagittal sinus. Lastly, the OC is unique among diploic vein pathways because its pathway is unpaired. It courses as a single dominant channel in the medial occipital bone superinferiorly, draining exrtacranially until it unites with the suboccipital venous channels. The OC had also been documented as having communication points with the confluence of sinuses.

In short, the calvarial diploic system articulates and communicates with a wide variety of intracranial dural sinuses as mentioned above. The major diploic vein pathways, PFP, FO, OP, and OC, develop in very heterogeneous ways among individuals, with added asymmetry [6].

Tsutsumi et al., [6] Garcia-Gonzales et al., [5] and Hershkovitz et al. [1] all characterized various classifications of the diploic veins to assorted extents. Further illustrating the diversity of anatomical variations and patterning that are present with diploic veins among individuals.

## Review

Vascular communication from the cortical surface and the meninges into the superior sagittal sinus could make the diploic veins a route of infection in specific cases. For example, sinusitis can lead to serious complications if the infection spreads to the intracranial space. Intracranial complications have been found to develop from direct extension from infected sinuses or hematogenous spread by the diploic veins [15].

As the diploë present such a great risk for spreading infection there is potential for more complex lymphatic communication across this space [5,16]. The CSF is filtered through arachnoid granulations that indent into the diploë [5]. The choroid plexus has antigen presentation capacity and can stimulate the proliferation of peripheral helper T lymphocytes via an Ia-dependent major histocompatibility complex (MHC) restricted mechanism. It is suggested that microglia and astrocytes here can present foreign antigens when lymphocyte levels within the central nervous system are low [17]. It is possible that there is greater communication between the dural venous sinuses and the general circulation than previously believed.

Tsutsumi et al. [18] demonstrated that diploic veins may provide an alternate flow for CSF drainage. Lipocalin-type prostaglandin D synthase (PGDS), a major endogenous β chaperone secreted in the CSF, and cystatin C (CysC), a low molecular weight cysteine proteinase inhibitor, are highly expressed in the CSF. Diploic veins have elevated PGDS and CysC, supporting CSF drainage through those veins. Contrast enhancement of diploic veins indicates that blood reaches the arterioles and capillaries of the dura mater before passing through venules and draining into the diploic veins [18].

Clinical significance

Arachnoid granulations act as outlets for intracranial CSF. Those in the cranial dural sinuses partner with diploic veins to drain CSF. Arachnoid pouches that extend into the skull communicate with contiguous diploic veins as an alternative route for CSF. T2-weighted images revealed diploic veins containing analogous hypersensitivity to that of CSF [6]. In a case report involving a 9-year-old male with a pseudomeningocele that extended into the diploic space, Bulleid et al. [19] proposed that the CSF was reabsorbed by the diploic space. This further indicates that the diploic space can act as an additional site for reabsorption of CSF, therefore causing an unwarranted rate of CSF reuptake [20]. As the diploic space serves as a site for CSF circulation, fistulas that occur here pose a high risk for potential meningitis and other infections. Placantonakis et al. [21] described two case reports involving CSF fistulas occurring post-neurosurgical procedures. One case in particular highlighted removal of the bone barrier during a routine craniectomy that ultimately caused the formation of an intradiploic CSF fistula because a thin cortical bone developed at the surgical site; furthermore, it failed to prevent the extravasation of CSF due to an increase in resistance of CSF. It is worth mentioning that the type of dural closure used in the initial operation, primary repair with hemostatic oxidized cellulose, failed to avert extravasation of CSF leading into the extradural compartment.

Bleeding from diploic veins can result in an acute subdural hematoma, which constitutes a medical emergency if not properly identified [22]. In a surgical case presented by Dodson et al. [22] involving a cochlear implantation resulted in an acute subdural hematoma due to excessive bleeding of the diploic veins. This particular study asserts that aging results in increased brittleness of the diploic veins and dura in response to shear forces.

Diploic arteriovenous fistulas are rare and have an unknown etiology, but when they occur they are generally traumatic [10,23]. Several hypotheses such as traumatic injuries, anatomical variations, and vascular pathologies (superior sagittal sinus thrombosis) can contribute to an environment suitable for the development of diploic arteriovenous fistulas [10]. The cause of concern with diploic arteriovenous fistulas is an atypical intraosseous anastomosis between diploic venous channels and meningeal arteries, which in principle could result in a greater incidence of bleeding [10].

## Conclusions

In a broad sense, the relationships of the diploic veins to emissary veins, dural sinuses, meninges, and scalp suggest a role in a wide range of pathologies related to either the CSF or venous circulation, such as thrombosis and cancer metastases. The diploic veins act as the link between the extra- and intra-cranial venous systems. The full extent of the connections of the diploic veins is not well understood, but with the extensive range of pathophysiological conditions related to the diploë it is imperative that their physiology be studied further.
